# Data of indirect immunofluorescence labeling of the mouse brain sections with sera from SLE and MS patients

**DOI:** 10.1016/j.dib.2017.09.027

**Published:** 2017-09-20

**Authors:** Ayse Ilksen Colpak, Banu Balci-Peynircioglu, Alp Can, Yasemin Gursoy-Ozdemir, Sevda Lule, Umut Kalyoncu, Turgay Dalkara

**Affiliations:** a Department of Neurology, Faculty of Medicine, Hacettepe University, Ankara, Turkey; b Institute of Neurological Sciences and Psychiatry, Faculty of Medicine, Hacettepe University, Ankara, Turkey; c Department of Histology and Embryology, School of Medicine, Ankara University, Ankara, Turkey; d Department of Internal Medicine, Division of Rheumatology, Faculty of Medicine, Hacettepe University, Ankara, Turkey; e Current affiliation: Department of Medical Biology, Faculty of Medicine, Hacettepe University, Ankara, Turkey; f Current affiliation: Department of Neurology, School of Medicine (KUSOM), Koç University, İstanbul, Turkey

**Keywords:** Indirect immunofluorescence, Multiple sclerosis, Systemic lupus erythematosus, Autoimmunity

## Abstract

The data presented in this article are related to the research article entitled “Behcet Disease serum is immunoreactive to neurofilament medium which share common epitopes to bacterial HSP-65, a putative trigger” (Lule et a. 2017) [1]. The immunoreactivity to self-antigens is well characterized for systemic lupus erythematosus (SLE) and multiple sclerosis (MS) (Magro Checa et al., 2013) [2]. Indirect immunofluorescence labeling of the mouse tissue sections with patient sera has recently been popular to discover novel epitopes and gain mechanistic insight to diseases with dysregulated immunity (Lennon et al., 2004) [3]. The present article demonstrates widespread labeling of cell nuclei with SLE patient sera and sporadic filamentous labeling along the axons with MS patient sera on mouse brain sections. The filamentous immunolabeling was sometimes associated with cytoplasmic staining of cells, which sent processes along the axon bundles, suggesting that they were oligodendrocytes. Since the mouse brain tissue has little autofluorescence and limited connective tissue causing non-specific immunolabeling, it appears superior to peripheral tissues for searching serum immunoreactivity.

**Specifications Table**

**Table:** 

Subject area	*Immunology*
More specific subject area	*Autoimmunity in Systemic Lupus Erythematosus and Multiple Sclerosis*
Type of data	*Images captured from brain slices incubated with patient sera*
How data was acquired	*Indirect immunofluorescence assay, confocal and fluorescent microscopy*
Data format	*Raw and analyzed*
Experimental factors	*Sera were obtained from patients with Systemic Lupus Erythematosus or Multiple Sclerosis and were investigated for their immunoreactivity against mouse brain tissue sections*
Experimental features	*Data illustrate the positive immunolabeling of mouse brain sections with patient sera exhibiting distinct patterns for each disease*
Data source location	*Hacettepe University, Ankara, Turkey*
Data accessibility	*The data are available in this article*
Related research article	*S. Lule, A. I. Colpak, B. Balci-Peynircioglu, Y. Gursoy-Ozdemir, S. Peker, U. Kalyoncu, A.Can, N. Tekin, D. Demiralp, T. Dalkara. Behcet Disease serum is immunoreactive to neurofilament medium which share common epitopes to bacterial HSP-65, a putative trigger. J Autoimmun. 2017.*

**Value of the data**

– SLE sera labeled numerous cell nuclei in cortical and subcortical areas in line with the presence of antinuclear antibodies in this disease.
– MS sera sporadically labeled cells (oligodendrocytes) and exhibited filamentous immunostaining along the axons in line with well-known immunoreactivity against myelin in MS
– These preliminary data suggest that indirect immunofluorescence labeling of the mouse brain sections with patient sera could be a simple assay to search for novel epitopes or gain mechanistic insight to known epitopes in SLE and MS.

## 1 Data

**Fig. 1:**
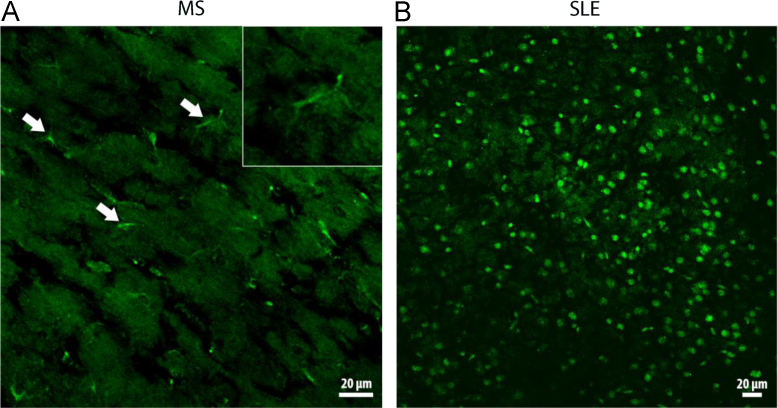
A and 1B: MS and SLE patient sera are immunoreactive against mouse brain sections.

The data presented in this manuscript have been generated in a study searching for a possible immunoreactivity of Behcet's Disease sera against mouse brain tissue [1]. The specificity of the immunoreactivity detected against the neurofilament-M protein in Behcet sera was tested by comparing it with immunoreactivity of sera from SLE and MS patients in addition to healthy controls. This disclosed that SLE and MS sera immunolabeled the mouse brain sections with distinct patterns specific for each disease (Fig. 1 A and 1B). Sera from all SLE patients caused widespread nuclear immunolabeling in brain sections, in line with the presence of anti-nuclear antibodies in SLE. Half of the MS sera labeled sporadic cells (some together with their processes) as well as filamentous structures along the axon bundles. It appears such that mouse brain sections that has little autofluorescence and connective tissue compared to peripheral tissues can be utilized to identify the possible epitopes in diseases such as SLE and MS. The non-specific binding of serum immunoglobulins to collagen and elastin complicates the evaluation of the specific binding in connective tissue rich tissues [4]. Thus, indirect immunofluorescence labeling of the mouse brain sections should be considered as an efficient, easy to use and inexpensive screening tool for discovering novel epitopes in autoimmune disorders compared to immunoblotting techniques and enzyme immunoassays such as enzyme-linked immunosorbent assay (ELISA) or multiplex immunoassays.

(A) Incubation of brain sections with sera from MS patients caused a filamentous immunolabeling along the axon bundles. Occasional cells exhibited cytoplasmic labeling extending into processes originating from the soma (arrows and inset). (B) Serum from a SLE patient extensively labeled cell nuclei, here shown in the cortex. Images were captured with a confocal microscope. Scale bars: 20 μm.

## 2 Experimental design, materials and methods

### 2.1 Patients and samples

SLE and MS sera were studied. Patients were recruited from neurology and rheumatology clinics of Hacettepe University Hospitals. The study was approved by the Ethics Committee of Faculty of Medicine, Hacettepe University, Ankara, Turkey (GO 14/361-05, FON 06/40-25). SLE patients were classified according to American College of Rheumatology revised criteria. MS patients were all relapsing- remitting type, on interferon-β1a or interferon-β1b treatment and blood samples were collected in steroid-free clinical remission period. Sera obtained from subjects were kept frozen at −80 °C until assayed. Freshly-isolated brain sections (20 μm) from Swiss albino mice were used for indirect immunofluorescence labeling.

Tissue sections were kept at −20 °C until immunolabeling with sera. Immunolabeling with sera was performed at room temperature (+23 to +25 °C).

### 2.2 Pre-absorption

Since serum samples contain many non-specific antibodies, preabsorption of patient and control sera with lyophilized guinea pig liver at 1/60 dilution in phosphate buffered saline (PBS) solution was performed as described by Lennon and co-workers [5] to minimize non-specific background staining. Pre-absorption was applied to all samples before immunostaining experiments.

### 2.3 Incubation of tissue sections with sera

For indirect immunofluorescence assay, 20 μm-thick, coronal mouse brain sections were cut on a freezing cryostat (Leica), fixed with 10% formaldehyde in PBS solution for 4 min and permeabilized with 1% CHAPS solution (AppliChem) in PBS. Sections were blocked with 10% normal goat serum (Millipore) at room temperature for 60 min and then incubated with pre-absorbed patient and control sera at 1/60 dilution (in PBS) for 70 min at room temperature. Secondary labeling was done with fluorescent-conjugated (FITC) goat anti-human IgG antibody (1/200 dilution, Jackson Immunoresearch) at room temperature for 60 minutes. Slides were washed 3 times for 5 min with PBS between the each step above in order to minimize non-specific background binding. After immunofluorescent labeling, brain sections were mounted with Hoechst solution (Hoechst 33258, Molecular Probes, 10 mg/ml in PBS) on microscope glass slides. Slides were evaluated under a wide-angle fluorescent (Nikon Eclipse E600) and confocal (Zeiss LSM-510) microscopes.
