# Supplementary material for: Data of indirect immunofluorescence labeling of the mouse brain sections with sera from SLE and MS patients
Source: Data Brief. 2017 Sep 20;15:170–3. doi: 10.1016/j.dib.2017.09.027 (PMC5633813; doi:10.1016/j.dib.2017.09.027)
Supplement: Supplementary file 1 — Supplementary material [file mmc1.docx]

Sep, 7, 2017

The authors declare that they have no conflict of interest.

Ayse Ilksen Colpak

Banu Balci-Peynircioglu

Alp Can

Yasemin Gursoy-Ozdemir

Sevda Lule

Umut Kalyoncu

Turgay Dalkara
